# Is Palivizumabe a Protective Factor for the Development of Asthma in Children? A Systematic Review With Metanalysis

**DOI:** 10.1002/ppul.71242

**Published:** 2025-08-22

**Authors:** Mariana Bueno Manini, Natali Caroline da Silva, Rafaela Agra de Castro, Milena Baptistella Grotta, Adyleia A. D. Contrera Toro

**Affiliations:** ^1^ Child and Adolescent Health Postgraduate Program State University of Campinas Campinas São Paulo Brazil; ^2^ Pediatric Pulmonology Medical Residency program at UNICAMP State University of Campinas Campinas São Paulo Brazil; ^3^ Center for Investigation in Pediatrics, Faculty of Medical Sciences State University of Campinas Campinas São Paulo Brazil; ^4^ Department of Pediatrics, School of Medical Sciences State University of Campinas Campinas São Paulo Brazil

**Keywords:** asthma, palivizumab, prematurity

## Abstract

**Introduction:**

Premature infants are at increased risk of developing chronic respiratory diseases, predisposing them to severe infections, such as those caused by respiratory syncytial virus (RSV). Palivizumab reduces the severity of RSV infections in high‐risk children; however, its relationship with asthma development in premature infants remains unclear.

**Objective:**

This systematic review with meta‐analysis aimed to review the literature and assess whether prophylaxis with palivizumab protects premature infants without congenital heart disease from developing asthma.

**Results:**

In total, 14 studies met the inclusion criteria, assessing 1,364,238 children; of these, 9232 received palivizumab. No significant difference in the chance of developing asthma between the groups (odds ratio (OR) of 0.84, 95% CI [0.62–1.13], *p* = 0.1968). Heterogeneity between studies was *I*² = 35.6%. Subgroup analysis for children with a family history of atopy showed no significant reduction in asthma risk (OR 0.78, 95% CI: 0.40–1.55, *p* = 0.3390). Sensitivity analysis confirmed result robustness. IgE levels were similar between the groups (standardized mean difference [SMD] −0.03 [95% CI: −0.30; 0.23], *p* = 0.8088). Children who received palivizumab were diagnosed younger (SMD −0.24 [95% CI: −0.38; −0.09], *p* = 0.0014), with lower gestational age (MD −0.75 [95% CI: −1.61; 0.12], *p* = 0.0915).

**Conclusions:**

Palivizumab prophylaxis does not reduce asthma risk in premature children without congenital heart disease. Its primary benefit lies in preventing severe RSV infections, with no direct impact on asthma developing.

## 1 Introduction

Premature children, especially extremely preterm infants, are more likely to develop chronic lung diseases [1]. Lung development begins during gestation and continues for years after birth [2, 3]. The alveolarization process is the final stage of respiratory system development, persisting throughout childhood and adolescence [4]. This process is often interrupted in premature infants, resulting in incomplete lung maturation, reduced alveolar number and size, and impaired lung capacity [3]. Airway formation also occurs early in fetal lung development, and premature newborns have reduced airway caliber [5]. Altered lung development, associated with an immature immune system, predisposes premature infants to an increased risk of severe respiratory infections, such as those caused by respiratory syncytial virus (RSV) [3].

Environmental factors like maternal smoking, early exposure to antibiotics, which negatively alters the microbiome, and cesarean delivery, which limits the transfer of protective microbiota from mother to baby, also increase the risk of respiratory diseases by impairing the development of the airways [6]. Severe RSV infections during the first 2 years of life have been associated with complications such as recurrent wheezing and asthma [7, 8]. However, the biological mechanism of this process has not been clearly explained [3, 4, 9].

Atopic asthma is characterized by an immunoglobulin E (IgE)‐mediated reaction accompanied by eosinophilic infiltration in the airways [10]. Evidence suggests that RSV infection may modulate the immune response, favoring Th2‐type response and decreasing IFN‐γ‐mediated antiviral immunity, thus contributing to airway hyperresponsiveness. This immunological mechanism seems to be relevant in the development of asthma in individuals with atopic predisposition and exposure to environmental factors [10, 11, 12]. Dysfunction of the epithelial barrier caused by viral infection allows the entry of allergens, maintaining the inflammatory cycle and contributing to an increased risk of developing asthma in children [11]. Factors such as age at first infection, viral coinfection, and genetic predisposition may act as modulators of this relationship. Although new preventive strategies, such as RSV vaccines and monoclonal antibodies, are being developed, their impact on reducing the risk of asthma at subsequent ages is still unknown [13].

Palivizumab, a humanized monoclonal antibody, was approved by the the Food and Drug Administration (FDA) in 1998 [14] and by Brazil′s Ministry of Health in 2013 [15, 16]. However, in the state of São Paulo, it has been used since 2007 [17]. It is a humanized monoclonal antibody that interacts with the F glycoprotein, located on the surface of RSV, inhibiting viral fusion with host cells [18]. It is used as prophylaxis to reduce severe RSV infection in high‐risk children, such as premature infants with or without bronchopulmonary dysplasia, with congenital heart disease with hemodynamic repercussions, and neuromuscular diseases [19]. A study that assessed premature children who received prophylaxis with palivizumab for 6 years showed no impact on the prevention of atopic asthma, but it prevented recurrent wheezing in these individuals up to the age of 6 years [20]. However, Simões et al. found that the use of passive immunization reduced the risk of recurrent wheezing (RW) only in children without a family history of atopy, suggesting that RSV predisposes to RW independently of the atopy [21]. Another study published in 2021 showed that 27.1% of premature children who received immunoprophylaxis against RSV developed recurrent wheezing. The authors concluded that genetic factors associated with atopy may be important predictors of RW [22]. The association between RSV infection and recurrent wheezing and asthma is clear; however, the cause‐and‐effect relationship has not been established and the pathophysiology has not been fully explained [6].

Therefore, this systematic review with meta‐analysis aimed to explore the scientific literature and determine whether prophylaxis with palivizumab in premature infants without heart disease reduces the incidence of asthma in preschoolers and schoolchildren. Understanding the factors involved in asthma development in this group may lead to better prevention and treatment strategies.

## 2 Methods

This study was conducted following the guidelines of the Preferred Reporting Items for Systematic Reviews and Meta‐Analyses (PRISMA) [23]. The study protocol was registered in PROSPERO in January 2021 (reference CRD42023404910). In addition, the guiding question of this review was designed to ensure a systematic search of the scientific literature using the PICO (Population, Intervention, Comparison, Outcomes) strategy [23].

### 2.1 Search Strategy

A systematic search was conducted in PubMed, Embase, BVS‐BIREME, Scopus, Web of Science, and the Cochrane Library to identify English‐language articles published until May 2024. The search terms used were: (“infant, premature”; AND palivizumab) AND (“infant, newborn” OR “baby” OR “preschool child” OR “child”) AND asthma. No year or filter limits were applied, and the search was restricted to the pediatric population. The search strategy for each database was developed in collaboration with a research librarian. The selected literature was managed using Rayyan [24], a web‐based tool designed to facilitate collaboration among reviewers during the study screening process. This tool allowed the import of studies from multiple databases, where titles and abstracts were displayed for screening. Assistant reviewers remained blind to the principal investigator′s decisions, enhancing the reliability and methodological rigor of the selection process.

The screening process was carried out in two phases: first, by title and abstract, and then through a full‐text review. Discrepancies were resolved by a third reviewer, who made the final decision on the inclusion or exclusion of studies. Duplicates were identified and removed, and a critical evaluation of the full‐text articles was conducted once all inconsistencies were resolved.

### 2.2 Inclusion and Exclusion Criteria

Studies were included in the systematic review if they met the following criteria: (1) randomized trials, observational studies (including cohort and case‐control studies), and secondary studies, (2) preterm children without heart disease who received palivizumab and were diagnosed with asthma up to 12 years of age, of all ethnicities and genders, (3) articles published in English, (4) articles with the following outcome: the use of palivizumab in preterm children without heart disease was or was not associated with the onset of asthma. The exclusion criteria were: (1) case reports and letters to the editor, (2) children over 12 years of age, animals or infants with heart disease, (3) articles in other languages, and (4) other outcomes.

### 2.3 Data Extraction and Risk of Bias Assessment

Data were extracted by two independent reviewers, and, in case of discrepancy, a third investigator made the final decision. For each eligible article, the following were recorded: authors, year of publication, country of study, study type, number of participants, age at asthma diagnosis, gestational age, objectives, asthma diagnostic methods, results, and outcomes. The risk of bias for each study was independently assessed by two reviewers using the Newcastle‐Ottawa Scale (NOS) [25], the Joanna Briggs Institute (JBI) tools [26], or the Cochrane RoB 2.0 [27].

### 2.4 Quantitative Analysis (Meta‐Analysis)

A meta‐analysis was performed to estimate the standardized mean difference between the groups and the respective confidence interval considering the random effects model. Summary measure was estimated with the metacont function of R package meta.

A meta‐analysis was performed to estimate the odds ratio of asthma occurrence between the groups and the respective confidence interval considering the random effects model. Summary measure was estimated with the metabin function of R package meta.

Cochran′s Q test and Higgins and Thompson′s I² statistic were used to assess the degree of heterogeneity between the studies. The results were grouped in a forest plot.

The sensitivity analysis was applied using the leave‐one‐out method for the main outcome (asthma). Meta‐regression was applied for the main outcome, adjusting for the prevalence of family history of atopy, using a mixed‐effect logistic regression model.

## 3 Results

**Figure 1:**
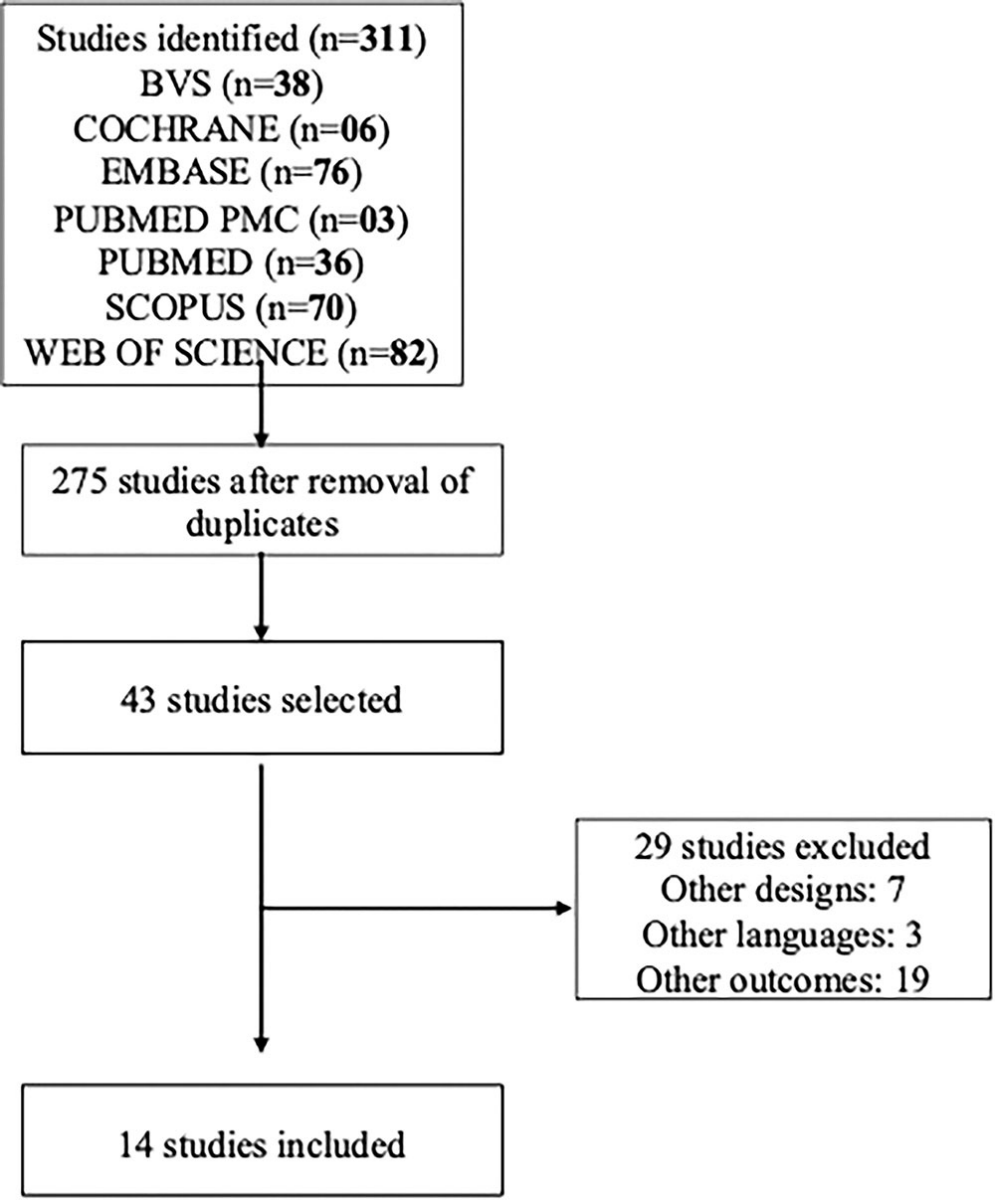
Flowchart of study selection.

During the literature review and search process, 311 studies were initially identified. After removing duplicates, 275 studies remained. Following title and abstract screening, 43 articles were selected for full‐text review. Detailed analysis resulted in the exclusion of 29 articles for the following reasons: different study design (*n* = 7), other language (*n* = 3) and the others did not present the desired outcome (*n* = 19). Then, 14 studies were considered eligible for this systematic review and meta‐analysis (Figure 1).

Of all 14 studies included, 10 were observational studies, two were cross‐sectional studies, and two were clinical trials. The studies were conducted in various countries: Israel (*n* = 2), the United States (*n* = 2), Taiwan (*n* = 1), Spain (*n* = 2), Denmark (*n* = 1), Sweden (*n* = 1), Turkey (*n* = 2), Japan (*n* = 2), Canada (*n* = 1), and the Netherlands (*n* = 2). Ten of them found no association between the use of palivizumab and asthma development, while four studies suggested a possible reduction in asthma risk. A total of 96,977 preterm children were included in the systematic review; of those, 8707 received immunoprophylaxis with palivizumab (Table 1) [28, 29, 30, 31, 32, 33, 34, 35, 36, 37, 38, 39, 40]. The assessment of the risk of bias showed that most studies were categorized as low risk, except for the study by Unal et al. [38], which was categorized as moderate risk due to the absence of satisfactory inclusion criteria, according to the JBI scale (Table 1).

**Table 1:** Characteristics of the studies included in the systematic review.

Author	Country	Type of study	Sample (*n*)	Gestational age (weeks)	Age at asthma diagnosis (years)	Asthma		Conclusion about Palivizumab	Risk of bias	
						I (%)	C (%)		Tool	Risk
Mochizuki et al. (2023) [20]	Japan/USA	Cohort study	268	33–35	6	31/202 (15.3%)	12/66 (18.2%)	Did not suppress the onset of atopic asthma	NOS	Low
Bar‐Yoseph et al. (2019) [28]	Israel	Cohort study	42	29–32	5–7	NI	NI	No beneficial effects observed in children	NOS	Low
Carroll et al. (2017) [29]	USA	Cohort study	6.566	< 35	4–6	1498/6566 (22.8%)	NI	It may not be effective in preventing asthma in children	NOS	Low
Garcia‐Garcia (2020) [31]	Spain	Cohort study	279	< 37	6–9	9/37 (24.3%)	NI	No significant association between its use and prevalence of asthma	NOS	Low
Haerskjold et al. (2017) [32]	Denmark and Sweden	Cohort study	84.004	21–36	0–4	NI	NI	Did not protect against asthma in the preterm group	NOS	Low
Igde et al. (2018) [33]	Turkey	Cohort study	226	24–40	2–5	7/113 (6.2%)	19/113 (16.8%)	Reduces the risk of asthma in children aged 2–5 years	NOS	Low
Jalink et al. (2019) [34]	Canada	Cohort study	3916	29–36	5	0	NI	There were no cases of asthma among subjects receiving the drug	NOS	Low
Morata‐Alba et al. (2019) [35]	Spain	Cohort study	116	32–35	6–8	NI	NI	Prophylaxis was a protective factor against obstructive spirometric pattern at 7–8 years of age	NOS	Low
Prais et al. (2016) [36]	Israel	Cross‐ sectional	63	< 29	7–10	10/30 (33.3%)	6/33 (18.2%)	No effect on long‐term airway responsiveness.	JBI	Low
Scheltema et al. (2018) [4]	Netherlands	Clinical trial	395	32–35	6	19/185 (10%)	18/182 (9.9%)	Did not have a major effect on current asthma or lung function.	Rob2	Low
Ünal et al. (2017) [38]	Turkey	Cross‐ sectional	98	< 37	2	NI	NI	Weak correlation with asthma	JBI	Moderate
Xu et al. (2021) [39]	Netherlands	Clinical trial	274	33–35	6	19/132 (14)	34/142 (24)	No direct link between epigenetic markers and asthma	Rob2	Low
Fang et al. (2023) [40]	Taiwan	Cohort study	576	23–37	5–20	101/306 (33%)	90/268 (33.5%)	May decrease asthma development and medication treatment time in children	NOS	Low
Kato et al. (2023) [28]	Japan	Cohort study	154	33–35	6	19/113 (17)	8/41 (19.5)	Was unable to suppress recurrent wheezing and the onset of asthma	NOS	Low

The studies included in the review used different clinical criteria for the diagnosis of asthma, such as clinical history and structured questionnaires. Some studies assessed allergy status through laboratory tests, such as serum IgE levels, skin prick tests, eosinophil count, and spirometry to assess lung function. These diagnostic tools are widely accepted and established in the literature as effective methods to identify asthma and assess the allergic response in patients (Table 2) [41, 42].

**Table 2:** Diagnosis of asthma.

Author	Definition of asthma	Laboratory tests
Igde et al. (2018) [33]	Modified asthma predicative index, a major criterion or two minor criteria.	Eosinophil count
Mochizuki et al. (2023) [20]	Recurrent wheezing with elevated serum total or specific IgE levels or family history of allergy.	IgE assay
Prais et al. (2016) [36]	Medical diagnosis of asthma.	Spirometry
Scheltema et al. (2018) [4]	Parent‐reported wheezing in the past 12 months or medication use in the past 12 months, or both. ISAAC questionnaire.	IgE assay; spirometry (FEV); FeNO
Xu et al. (2021) [40]	Parent‐reported wheezing or asthma medication use in the past 12 months.	IgE assay; spirometry (FEV)
Fang et al. (2023) [39]	Medical diagnosis of asthma with prescription of associated medications in the past 12 months.	IgE assay
Kato et al. (2023) [28]	Modified ISAAC questionnaire.	IgE assay
Bar‐Yoseph et al. (2019) [29]	Methacholine challenge test (PC_20_).	FeNO; methacholine challenge test; inflammatory cytokine levels; spirometry
Carroll et al. (2017) [30]	Medical diagnosis of asthma and prescription of asthma medications.	—
Garcia‐Garcia (2020) [31]	ISAAC questionnaire and medical diagnosis of asthma.	Spirometry; skin prick test
Haerskjold et al. (2017) [32]	Medical diagnosis of asthma and use of medications	—
Jalink et al. (2019) [34]	Medical diagnosis.	—
Morata‐Alba et al. (2019) [35]	ISAAC questionnaire and medical diagnosis.	FeNO
Ünal et al. (2017) [38]	Questionnaire, wheezing, shortness of breath or coughing fits lasting more than 24 h, at least 2 to 3 times a year.	IgE; skin prick test

**Figure 2:**
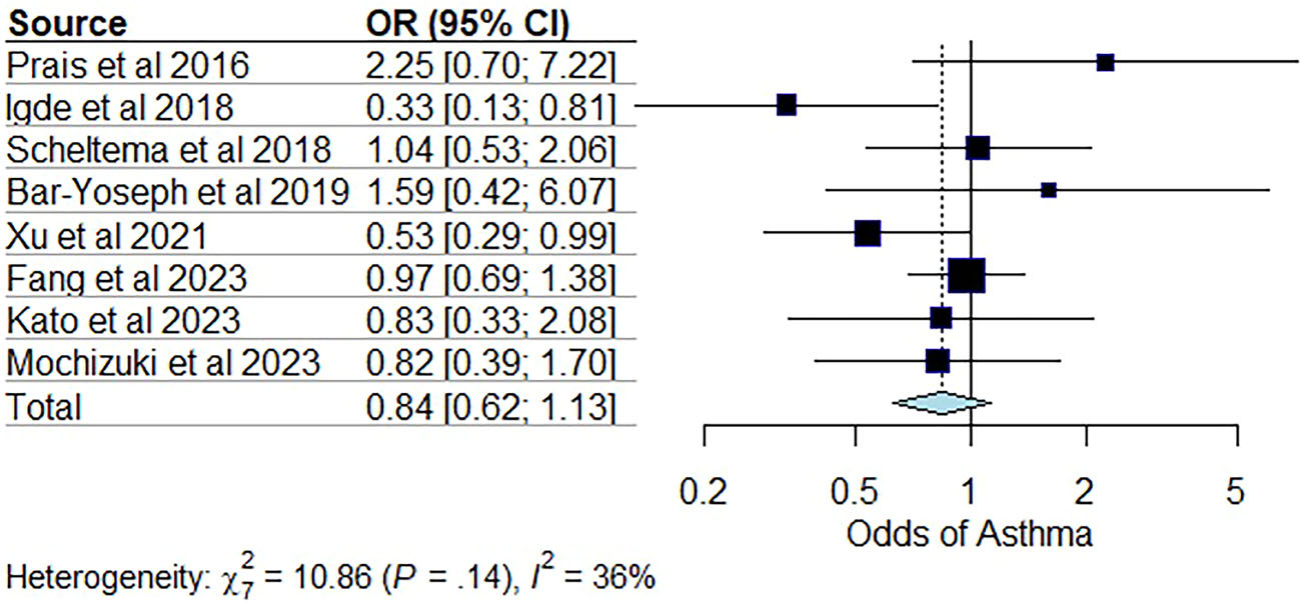
Results of the meta‐analysis to estimate the chance of asthma between groups. [Color figure can be viewed at wileyonlinelibrary.com]

In the meta‐analysis to estimate the chance of asthma between the groups treated and not treated with palivizumab, 1965 subjects were included, of which 1107 received prophylaxis with palivizumab. This analysis was possible after the exclusion of six articles [4, 20, 33, 36, 39, 40] that did not provide sufficient data on the number of individuals who received immunoprophylaxis and developed asthma, making this type of statistical analysis unfeasible. The pooled odds ratio (OR) was 0.84 (95% CI [0.62; 1.13]), suggesting a 16% reduction in the chance of developing asthma in children who received palivizumab when compared to those who did not. However, this reduction was not statistically significant (*p* = 0.2519). Heterogeneity between studies was I^2^ = 35.6%. Meta‐regression was performed to adjust the risk of developing asthma due to family history of atopy in five articles [20, 28, 29, 33, 36]. Subgroup analysis adjusting for family history of atopy resulted in an adjusted OR of 0.78 (95% CI [0.40–1.55], *p* = 0.3390), indicating a potential 22% reduction in asthma risk, though this finding lacked statistical significance. Sensitivity analysis confirmed that excluding individual studies did not substantially alter the results, highlighting the robustness of the meta‐analysis findings (Figure 2).

**Figure 3:**
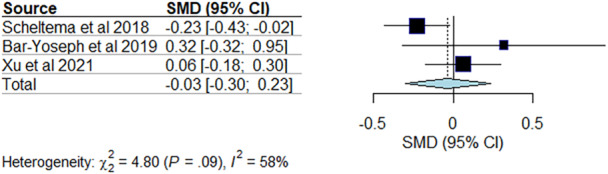
Results of the meta‐analysis to estimate the standardized mean difference for IgE between groups. [Color figure can be viewed at wileyonlinelibrary.com]

Regarding IgE levels, there was no significant difference between the groups, with a standardized mean difference (SMD) of −0.03 (95% CI: −0.30; 0.23, *p* = 0.8088). The analysis showed moderate heterogeneity, with I² = 58.3%, (*p* = 0.0909) (Figure 3).

**Figure 4:**
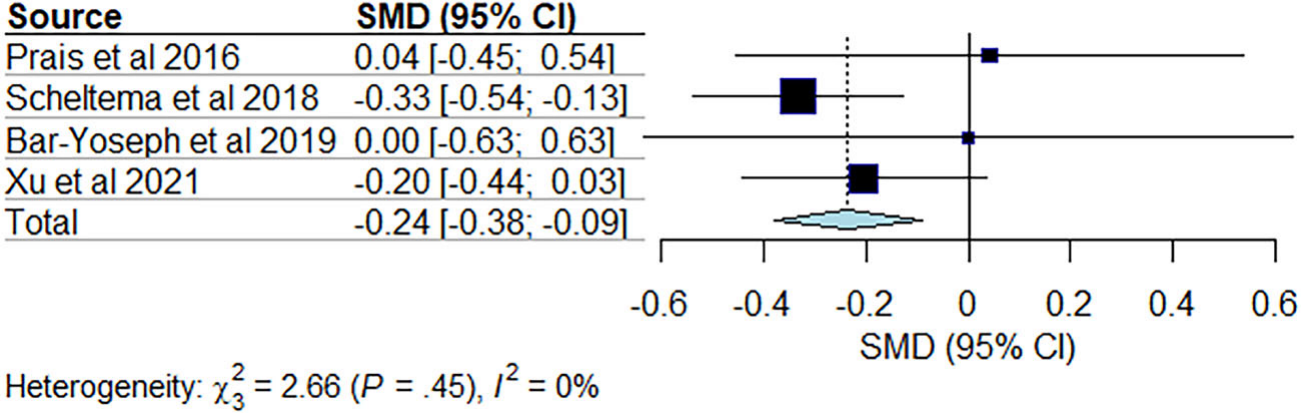
Results of the meta‐analysis to estimate the standardized mean difference for age at diagnosis between the groups. [Color figure can be viewed at wileyonlinelibrary.com]

The age at asthma diagnosis was significantly younger in children who received palivizumab (SMD −0.24 [95% CI: −0.38; −0.09], *p* = 0.0014) (Figure 4)

Gestational age was lower in the palivizumab group, but the difference was not statistically significant.

## 4 Discussion

In this systematic review with meta‐analysis, no association was found between the use of palivizumab and the subsequent development of asthma in preterm children without congenital heart disease.

The relationship between prophylaxis with palivizumab and the development of asthma in children has been widely investigated, with inconsistent results and conclusions. The analysis of the 14 studies included in this review demonstrates the complexity of this relationship. Ten studies found no evidence of protection against the development of asthma in preschool and school‐aged children. On the other hand, four studies suggested a possible reduction in asthma risk following the use of palivizumab. However, these results were not uniformly replicated, which underscores the need for caution in interpretation [33, 38, 39].

Severe RSV infection is recognized as a risk factor for the development of asthma in childhood. The use of monoclonal antibodies, by reducing the severity of this infection, has not consistently demonstrated a reduction in the risk of developing atopic asthma in preterm children. Additionally, methodological variability among studies contributes to divergent conclusions. Differences in the diagnostic criteria for asthma, from physician diagnoses based on spirometry to parental reports through standardized questionnaires, can introduce biases and limit the comparability of results.

In the articles selected for meta‐analysis, we observed a protective trend with a 16% reduction in asthma risk without adjustment and 22% with adjustment for a family history of atopy. However, these results did not show statistical significance, and we cannot conclude that palivizumab prevents asthma in children, regardless of genetic predisposition. Studies show that a family history of atopy increases the likelihood of developing asthma due to genetic predisposition [43, 44]. The absence of severe infection can reduce the likelihood of developing asthma, especially in children without a family history of atopy, as observed in some individual studies [33, 35, 38, 39]. However, when adjusting for the presence of familial atopy, the efficacy of palivizumab as a protective factor is reduced, since these children are predisposed to developing asthma regardless of viral infections.

The meta‐analysis results indicate that there was no significant difference in IgE levels between children who received palivizumab and those who did not. This is consistent with the hypothesis that although palivizumab prevents severe RSV infections, it does not directly affect the atopic sensitization process [45]. The IgE‐mediated allergic response is related to genetic and environmental factors [45], which may not be influenced using palivizumab. Therefore, palivizumab may not impact the developmental pathway of IgE‐mediated atopy. The lack of a protective effect against asthma may be attributed to the complex and multifactorial nature of the disease, in which genetic factors often outweigh the influence of isolated viral infections [12, 43, 44]. In individuals with atopic predisposition, activation of the Th2 immune response may occur independently of RSV infection, while epigenetic changes induced by diverse environmental exposures can perpetuate asthma risk [10, 40]. Since palivizumab acts exclusively by neutralizing RSV, without interfering with these underlying immunological and epigenetic mechanisms, its limitations in preventing asthma become evident [11, 37]. In this context, understanding these mechanisms becomes increasingly relevant in light of the potential replacement of palivizumab by broader and longer‐lasting prophylactic strategies, such as vaccines or extended half‐life monoclonal antibodies, which may exert a more comprehensive effect on antiviral immunity and inflammatory responses [5, 13, 19]. These findings reinforce that palivizumab acts as a protective factor mainly in the prevention of severe RSV infections, without modulating allergic immune responses, such as elevated IgE.

Children who received the monoclonal antibody were diagnosed earlier than the control group. Diagnosing asthma in children under five presents challenges, mainly due to the lack of specific diagnostic tests and the overlap of symptoms with other common respiratory diseases in this age group [46].

Prematurely born children are at increased risk of respiratory complications. In this study, the lower gestational age in the group that received palivizumab did not have an impact as a risk factor for asthma. Gestational age was a key inclusion criterion in this review, and only studies involving preterm infants were included. Despite some variability across studies—likely reflecting differences in national guidelines for palivizumab eligibility—gestational age was generally balanced between exposed and unexposed groups. This minimizes the risk of systematic bias. Although subgroup analyses by gestational age strata were not consistently available, clinical characteristics across studies were similar, and no clear imbalance was observed. In light of this, we believe gestational age did not significantly influence the observed associations.

Therefore, based on the results of this review, the use of palivizumab does not demonstrate a protective effect against the development of asthma. Asthma is a complex condition resulting from the interaction between genetic predisposition and environmental exposures, which trigger an inflammatory process in the airways and subsequent pulmonary complications.

In the analyzed literature, a variety of diagnostic criteria for asthma were observed. Diagnosing asthma in children is complex due to the variability of phenotypes, requiring the identification of a pattern of respiratory symptoms associated with variable expiratory airflow limitation, confirmed through pulmonary spirometry and a positive response to a bronchodilator [47]. In addition to characteristic clinical symptoms, asthmatic patients commonly have a personal or family history of atopy [12]. However, a significant number of individuals with asthma do not have any family history, highlighting that this condition results from the interaction between genetic predisposition and certain environmental exposures [12].

Additionally, children who received palivizumab had lower gestational age, but this was not statistically significant, and we cannot confidently conclude that palivizumab impacts this variable, despite the high variability observed among studies. Independent studies with subgroups of preterm infants are needed, given the differences in pulmonary development and immune response maturity. The use of more rigorous methodologies in future studies should provide answers to better understand the true role of palivizumab in asthma prophylaxis.

To date, no other meta‐analysis explores the association between the use of palivizumab and the development of asthma in preschool and school‐aged children, bringing original results to this study. However, there are limitations to this review, such as the lack of data standardization among the included studies and the absence of representation from studies conducted in continents such as South America, Africa, and Oceania, where legislation and risk factors may vary. Methodological improvement and the inclusion of more diverse populations in future studies are important to understand the disease′s etiology and prevention.

## 5 Conclusions

Palivizumab did not show a clear and consistent protective effect against asthma risk when analyzed collectively with all studies included in the meta‐analysis. The protection conferred by palivizumab is related to preventing severe RSV infections, without a direct impact on reducing the risk of asthma development in preterm children without congenital heart disease.

## Author Contributions

**Mariana Bueno Manini:** conceptualization, supervision, data curation, investigation, methodology, writing – review and editing, writing – original draft, visualization, project administration, formal analysis. **Natali Caroline da Silva:** formal analysis, investigation, writing – original draft, writing – review and editing, data curation. **Rafaela Agra de Castro:** investigation, writing – original draft, writing – review and editing, formal analysis, data curation. **Milena Baptistella Grotta:** writing – review and editing. **Adyleia A. D. Contrera Toro:** conceptualization, writing – review and editing, supervision; formal analysis.

## Conflicts of Interest

The authors declare no conflicts of interest.

## Data Availability

The data that support the findings of this study were derived from publicly available articles included in the systematic review. No new data were generated or analyzed during the current study.
